# Synergistic prognostic value of transcription factor multiplicity and Ki-67 in pituitary neuroendocrine tumour recurrence after endoscopic transsphenoidal surgery: a novel tiered molecular risk model

**DOI:** 10.3389/fendo.2026.1809624

**Published:** 2026-08-06

**Authors:** Dan Taracha, Hui Ma, Bravina Mary Nanyama, Zhaohui Wang, MST Israt Jahan Oshru, Eric Nziza, Bilal Ali, Hanmeng Guo

**Affiliations:** 1 Neurosurgery Department, General Hospital of Ningxia Medical University, Yinchuan, China; 2 Ningxia Medical University, Yinchuan, China; 3 Surgery Department, Bungoma County Referral Hospital, Bungoma, Kenya; 4 Kakamega County General Teaching and Referral Hospital, Kakamega, Kenya

**Keywords:** Ki-67, pituitary neuroendocrine tumours (PitNETs), prognostic model, recurrence, risk stratification, synergistic molecular interaction, transcription factors (SF-1, T-PIT, PIT-1)

## Abstract

**Background:**

WHO 2022 classification underscores lineage-defining transcription factors (TFs) as key determinants of pituitary neuroendocrine tumour (PitNET) biology. We hypothesise that interaction between lineage “driver” (TFs) and proliferative “engine” (Ki-67) provides superior prognostic power for predicting recurrence compared to either factor in isolation or to traditional clinicopathological parameters.

**Materials and methods:**

This retrospective cohort study analysed 302 patients who underwent primary Endoscopic Endonasal Transsphenoidal Surgery for PitNETs. Five prognostic models were constructed to simulate diverse pathology laboratory capabilities for global utility: specific TF/Ki-67 combinations (Model A), specific TFs only (B), unspecific TF multiplicity/Ki-67 (C), unspecific TF multiplicity only (D), and Ki-67 alone (E). Multivariate Cox regression, adjusted for key clinical confounders, assessed associations with recurrence.

**Results:**

Eighty-one patients (26.82%) experienced recurrence, median follow-up: 30 months (range: 2–69). Model A & C demonstrated superior prognostic performance, identifying multiple high-risk profiles (adjusted HR [adj.HR] range: 5.36-17.70, all P<0.05). The practical Model C performed nearly equivalently to the granular Model A. Their findings were synthesised into simplified 4-tiered molecular risk stratification. Using Tier I as reference, adj.HR for recurrence escalated significantly across tiers: Model A, Tier II (adj.HR=3.65, P = 0.081), Tier III (adj.HR=6.65, P = 0.009), Tier IV (adj.HR=15.44, P = 0.001); and Model C, Tier II (adj.HR=3.47, P = 0.097), Tier III (adj.HR=6.50, P = 0.010), Tier IV (adj.HR=17.78, P = 0.002). In models lacking Ki-67, only SF-1(+) & T-PIT(+) (Model B, adj.HR=2.75, P = 0.037) and triple-TF positivity (Models B & D, adj.HR~3.80, P = 0.012) retained significance. Model E, Ki-67 ≥3% (adj.HR=1.76, P = 0.015) remained significant.

**Conclusion:**

This study validates critical prognostic synergy between TF multiplicity and elevated Ki-67 in PitNET recurrence. We propose a novel tiered molecular risk model anchored in this interaction. Whilst this synergy is best captured by the comprehensive Model A, the full spectrum of models (A–E) provides a flexible diagnostic framework. This ensures risk stratification is accessible to pathology services with varying technical capabilities, to guide personalised post-operative management and surveillance.

## 1 Introduction

The fifth edition World Health Organization (WHO) classification formalises the term pituitary neuroendocrine tumour (PitNET), reflecting their potential for aggressive behaviour and recurrence (1, 2). Despite advances in endoscopic transsphenoidal surgery, recurrence remains a formidable challenge in long-term management (3, 4). It imposes a profound burden, leading to debilitating symptoms, complex re-operations, and significant psychosocial and socioeconomic strain on patients, their families, and healthcare system (5–7).

Predicting recurrence is therefore critical. Extensive research has identified numerous clinical, radiological, and surgical predictors of recurrence (8–10). However, single-factor models have limited predictive accuracy, as the biological behaviour of pituitary neuroendocrine tumours (PitNETs) is highly heterogeneous (11, 12). A more integrated, biologically grounded approach is needed to better capture this complexity and improve prognostication (13).

The WHO 2022 classification provides this essential framework. It emphasises that lineage-defining transcription factors (TFs), PIT-1, SF-1, and T-PIT, are the key determinants of intrinsic tumour biology (1, 2, 14). Concurrently, Ki-67 proliferative index is a cornerstone marker of aggressive potential, with a threshold of 3% widely used in clinical prognostication (12, 15). TFs and Ki-67 therefore provide a biologically relevant framework for assessing prognosis. However, the predictive power of Ki-67 is not absolute; a high index does not invariably lead to recurrence, and a low index does not guarantee cure (12). A tumour with a Ki-67 of 2% may recur aggressively whilst another with 5% may remain quiescent for years. This paradox reveals the inadequacy of isolated markers and suggests that proliferative potential must be interpreted within its specific molecular context, defined by TF expression (11, 16).

We hypothesise that a tumour’s behaviour is dictated by synergy between its lineage-defining TF (the “driver”) and its Ki-67 proliferative index (the “engine”) (1, 17). The distinct biological pathways activated by a specific TF may profoundly modify the oncogenic impact of high proliferation (14, 16, 18). Furthermore, a subset of tumours demonstrates multi-lineage differentiation (co-expression of two or more TFs), which is increasingly recognised and suggests a less differentiated, more aggressive biology that remains poorly understood (19–21).

Currently, most prognostic studies analyse TFs and Ki-67 as separate covariates, overlooking their potential interaction (9, 15, 22). This represents a significant knowledge gap, as synergistic models could provide superior risk stratification (13, 23). To address this, we first established a robust clinical-pathological prognostic model in our cohort [Part 1- Taracha D, Ma H, Yourui Z, Yang L, Nanyama BM, Ngouvi F, et al. A Clinical-Pathological Nomogram Incorporating WHO 2022 Subtypes for Individualized Prediction of Pituitary Neuroendocrine Tumor Recurrence After Endoscopic Transsphenoidal Surgery. *BMC Endocr Disord*. 2026; Manuscript under review, Submission ID: d8dae5e2-f498-4649-bea4-36b4a2c4c4dd (companion study)]. Building on that foundation, this study (Part 2) aims to bridge the biological gap by investigating synergistic interaction between TF profiles and Ki-67 status.

Critically, global pathology capabilities vary significantly. Limited antibody panels, expertise, and other resource constraints can impede ability of some laboratories to perform definitive TF subtyping and/or assess Ki-67 (2, 24). To ensure broad clinical utility, we developed five distinct prognostic models (A-E) to simulate a spectrum of pathology laboratory capabilities, ranging from centres performing full WHO 2022 molecular subtyping to those assessing only Ki-67 or basic TF multiplicity. This approach allows us to rigorously test our core biological hypothesis whilst providing a flexible, practical framework for molecular risk stratification adaptable to diverse clinical settings.

## 2 Materials and methods

### 2.1 Study design and ethical approval

This retrospective cohort study was conducted at the General Hospital of Ningxia Medical University. The institutional ethics committee approved the study protocol and granted a waiver for informed consent due to the retrospective design. All patients had provided written consent for surgery. The study adhered to the 1964 Declaration of Helsinki and its later amendments, and reported following STROBE (Strengthening the Reporting of Observational Studies in Epidemiology) guidelines.

### 2.2 Patient selection

This study utilises the same patient cohort and foundational clinical data as described in our companion clinical-pathological analysis (Part 1, under review, Submission ID: d8dae5e2-f498-4649-bea4-36b4a2c4c4dd). We reviewed all consecutive patients who underwent primary endoscopic endonasal transsphenoidal surgery (EETS) for PitNETs between January 2020 and September 2024. This timeframe ensured availability of WHO 2022-standard immunohistochemistry.

Inclusion criteria were: (1) histopathological confirmation of a PitNET; (2) primary resection via Endoscopic Endonasal Transsphenoidal Surgery (EETS) at our centre; (3) a minimum follow-up of 12 months; and (4) complete clinical and molecular (Transcription Factor and Ki-67) data.

Exclusion criteria were: (1) prior pituitary surgery or radiotherapy; (2) concurrent intracranial malignancy; (3) major intraoperative complications interfering with resection; (4) adjuvant therapy before confirmed recurrence; (5) incomplete follow-up (less than 12 months); (6) surgical approach other than EETS or (7) severe impairment of vital organs. After applying these criteria, 302 patients were included in the study.

### 2.3 Data collection

Data extraction from the Hospital Information System was concluded in September 2025. Two independent clinicians performed data entry with verification. Collected variables included demographics, presenting symptoms, comorbidities (diabetes mellitus, hypertension), and lifestyle factors. Radiological parameters included tumour size, modified Knosp grade (25), and invasion of the clivus or sphenoid sinus. Surgical records provided data on extent of resection (radical vs. partial) (26, 27), tumour consistency, vascularity, and cerebrospinal fluid (CSF) leakage.

### 2.4 Pathological assessment and immunohistochemistry

All tumour specimens underwent standardised immunohistochemistry (IHC) for transcription factors (PIT-1, SF-1, T-PIT), anterior pituitary hormones, and Ki-67. Two dedicated pituitary pathologists, blinded to clinical outcomes, interpreted all slides. Ki-67 labeling index was dichotomised at the clinically relevant threshold of <3% vs. ≥3% (12, 15). Tumours were classified according to the WHO 2022 criteria, based on transcription factor expression, hormone profiles, and morphological features (1, 2).

### 2.5 Definition of synergistic molecular variables

To investigate interactions and ensure global utility to different clinical settings, we constructed five distinct prognostic models to simulate varying diagnostic scenarios. Each model comprises a set of exhaustive and mutually exclusive categorical variables.

- Model A (Specific TFs + Ki-67): A 16-category variable combining each specific TF profile (single, dual and triple positivity for PIT-1, SF-1, and T-PIT, as well as negativity for all) with Ki-67 status (<3% or ≥3%).
- Model B (Specific TFs only): A 6-category variable based on specific TF combinations (All TFs(-); PIT-1 & SF-1; PIT-1 & T-PIT; SF-1 & T-PIT; and triple positivity), excluding Ki-67.
- Model C (Non-specific TF Multiplicity + Ki-67): An 8-category variable based on the number of positive TFs (0, 1, 2, or 3) regardless of which specific TFs are expressed, combined with Ki-67 status.
- Model D (Non-specific TF Multiplicity only): A 4-category variable based solely on the number of positive TFs (0, 1, 2, or 3) regardless of which specific TFs are expressed.
- Model E (Ki-67 only): A 2-category variable (Ki-67 <3% vs. ≥3%).

For all analyses, the reference category was defined as the biologically least aggressive profile: All TFs(-) & Ki-67<3%, for Models A and C, All TFs(-) for Models B and D, and Ki-67<3% for Model E. (All TFs(-): all three transcription factors are negative).

### 2.6 Outcome definition and follow-up

The primary endpoint was tumour recurrence. Either radiological or biochemical evidence qualified as recurrence. Radiological recurrence was defined as appearance of a new enhancing lesion after prior complete resection, or an increase of ≥20% in maximum diameter of a known residual lesion on serial contrast-enhanced MRI (3, 4, 9). Biochemical recurrence was defined as sustained hormone hypersecretion surpassing postoperative baseline or previously normalised levels in functioning tumours (9).

Patients had a routine follow-up protocol; they were followed until tumour recurrence or censoring at their last clinical visit (median follow-up: 30 months; range: 2–69). Clinical assessments, MRI, and relevant hormone evaluations were performed at 1–3, 6, and 12 months post-operatively, then annually (27). Time-to-recurrence was calculated in months from date of surgery to date of recurrence confirmation. Patients without recurrence were censored at their last follow-up visit. The study closed on September 2025.

### 2.7 Statistical analysis

Analyses were performed using IBM SPSS Statistics (Version 27.0) and R software (Version 4.3.2). A two-sided P-value <0.05 indicated statistical significance.

Cox proportional hazards regression: Univariate Cox analyses were performed to assess association of each synergistic variable within Models A–E with recurrence.

To test independent prognostic value of the molecular variables, we pre-specified three significant covariates from our prior clinical analysis (Part 1) [extent of resection (partial vs. radical), clival invasion (yes/no), and diabetes mellitus (yes/no)] as mandatory adjustment factors in all subsequent multivariate models (23). Multivariate Cox regression was conducted for each molecular model (A-E) whilst adjusting for these three clinical confounders. Results were presented as adjusted hazard ratios (adj.HR) with 95% confidence intervals (CI).

Development of tiered risk stratification: Based on the pattern of significant adjusted HRs, synergistic groups from the high-performing Models A and C were consolidated into simplified 4-tiered molecular risk stratification (Tier I: Low Risk to Tier IV: Very High Risk). Association of these tiers with recurrence was evaluated using adjusted Cox regression, following the principle of creating clinically useful risk categories (28, 29).

Model validation: Internal validation was performed using 500 bootstrap resamples to correct for optimism. Discrimination was assessed using Harrell’s C-index, with values above 0.60 considered acceptable/moderate, values above 0.70 considered good and values above 0.80 considered excellent. Calibration was evaluated visually using calibration plots at 1, 3, and 5 years, and quantitatively by calibration slope (ideal = 1.0). Time-dependent area under the curve (AUC) analysis was performed to further assess discrimination at 1, 3, and 5 years.

Survival analysis: Recurrence-free survival (RFS) was visualised using Kaplan-Meier curves, with comparisons made using Log-rank test. A forest plot was generated to summarise final multivariate results.

## 3 Results

### 3.1 Cohort characteristics

A total of 302 patients who underwent primary EETS for PitNETs between January 2020 and September 2024 were included. The cohort comprised 166 males (55.0%) and 136 females (45.0%), with a mean age of 51.5 ± 12.7 years. Tumour recurrence occurred in 81 patients (26.8%) over a median follow-up of 30 months (range: 2–69). **Table 1**. Demographic and baseline clinical characteristics of the included patients.

**Table 1:** Baseline characteristics.

Variable	No recurrence (221)	Recurrence (81)	Total (302)
Male/Female	125/96	41/40	166 (55.0%)/136 (45.0%)
Age (years), Mean ± SD	51.8 ± 12.6	50.7 ± 13.1	51.5 ± 12.7
<50/50 – 64/≥ 65	83/107/31	35/32/14	118 (39.1%)/139 (46.0%)/45 (14.9%)
Follow-up, Months, Median (Range)	33 (12-60)	15 (2-69)	30 (2-69)
Lifestyle factors			
BMI, Median (IQR)	24.8 (22.1-27.2)	25.2 (22.1-27.6)	24.9 (22.2-27.4)
BMI (<25Kg/m2/≥25Kg/m2)	115/106	39/42	154 (51.0%)/148 (49.0%)
Smoking (Y/N)	49/172	14/67	63 (20.9%)/239 (79.1%)
Alcohol (Y/N)	26/195	6/75	32 (10.6%)/270 (89.4%)
Presenting symptoms			
Visual Disturbance (Y/N)	145/76	53/28	198 (65.6%)/104 (34.4%)
Headache (Y/N)	112/109	43/38	155 (51.3%)/147 (48.7%)
Dizziness (Y/N)	91/130	33/48	124 (41.1%)/178 (58.9%)
Incidental Finding (Y/N)	39/182	11/70	50 (16.6%)/252 (83.4%)
Acromegaly (Y/N)	27/194	14/67	41 (13.6%)/261 (86.4%)
Menstrual Disorders (Y/N)	24/197	11/70	35 (11.6%)/267 (88.4%)
Body Weakness (Y/N)	24/197	5/76	29 (9.6%)/273 (90.4%)
Changes in Sexual Function (Y/N)	13/208	4/77	17 (5.6%)/285 (94.4%)
Lactation (Y/N)	4/217	0/81	4 (1.3%)/298 (98.7%)
Comorbid conditions			
Hypertension (Y/N)	60/161	23/58	83 (27.5%)/219 (72.5%)
Diabetes Mellitus (Y/N)	32/189	25/56	57 (18.9%)/245 (81.1%)
Coronary Heart Disease (Y/N)	9/212	7/74	16 (5.3%)/286 (94.7%)
Tumour characteristics			
Giant Macroadenoma/Non-Giant Macroadenoma	68/153	41/40	109 (36.1%)/193 (63.9%)
Sphenoid Sinus Invasion (Y/N)	78/143	42/39	120 (39.7%)/182 (60.3%)
Clival Invasion (Y/N)	37/184	32/49	69 (22.8%)/233 (77.2%)
Knosp Grade (0–2/3A–4)	128/93	36/45	164 (54.3%)/138 (45.7%)
Pituitary Apoplexy (Y/N)	97/124	33/48	130 (43.0%)/172 (57.0%)
Optic Chiasm Compression (Y/N)	162/59	62/19	224 (74.2%)/78 (25.8%)
Tumour Vascularity (Normal/Hyper-vascular)	133/88	44/37	177 (58.6%)/125 (41.4%)
Tumour Consistency (Soft/Firm)	154/67	54/27	208 (68.9%)/94 (31.1%)
Gross Tumour Color (Gray-white/Gray-red/Dark-red/Red/Dark-brown/White/Yellow)	175/20/10/7/5/3/1	64/9/7/1/0/0/0	239 (79.1%)/29 (9.6%)/17 (5.6%)/8 (2.6%)/5 (1.7%)/3 (1.0%)/1 (0.3%)
Surgical factors			
Extent of Resection (Radical/Partial)	151/70	34/47	185 (61.3%)/117 (38.7%)
Residual Tumour (Y/N)	170/51	67/14	237 (78.5%)/65 (21.5%)
CSF Leakage (Y/N)	36/185	7/74	43 (14.2%)/259 (85.8%)
Duration of Surgery (minutes)	200.5 ± 52.9	234.8 ± 95.2	209.7 ± 68.4
Ki-67 (≥3%/<3%)	66/155	43/38	109 (36.1%)/193 (63.9%)
WHO 2022 subtype			
Unclassified Plurihormonal Tumour	57	29	86 (28.5%)
Gonadotroph Tumour	43	14	57 (18.9%)
Null Cell Tumour	38	9	47 (15.6%)
Sparsely Granulated Corticotroph Tumour	20	8	28 (9.3%)
Densely Granulated Corticotroph Tumour	23	2	25 (8.3%)
Densely Granulated Somatotroph Tumour	16	2	18 (6.0%)
Sparsely Granulated Somatotroph Tumour	2	5	7 (2.3%)
Densely Granulated Lactotroph Tumour	5	2	7 (2.3%)
Thyrotroph Tumour	3	2	5 (1.7%)
Mammosomatotroph Tumour	4	1	5 (1.7%)
Acidophil Stem Cell Tumour	3	1	4 (1.3%)
Crooke Cell Tumour	2	2	4 (1.3%)
Sparsely Granulated Lactotroph Tumour	3	1	4 (1.3%)
Immature PIT1-Lineage Tumour	1	2	3 (1.0%)
Mature PIT1-Lineage Tumour	1	1	2 (0.7%)

SD, Standard Deviation.

IQR, Interquartile range.

Y/N, Yes/No.

BMI, Body mass index.

CSF, Cerebrospinal fluid.

Ki-67, Ki-67 labelling index.

WHO, World Health Organization.

Distribution of individual lineage-defining transcription factors was as follows: PIT-1 positive in 116 tumours (38.4%), SF-1 in 106 (35.1%), and T-PIT in 131 (43.4%). Ki-67 ≥3% was more prevalent in the recurrence group (53.1%) than in the non-recurrence group (29.9%). Distribution of patients across individual transcription factors and synergistic models (A-E) stratified by recurrence status is presented in **Table 2**.

**Table 2:** Individual transcription factors and synergistic models (A-E) stratified by recurrence status.

Variable name	No recurrence (N = 221)	Recurrence (N = 81)	Total (N = 302)
Individual transcription factors:			
PIT-1	79 (35.7%)	37 (45.7%)	116 (38.4%)
SF-1	73 (33.0%)	33 (40.7%)	106 (35.1%)
T-PIT	94 (42.5%)	37 (45.7%)	131 (43.4%)
Model A:			
All TFs(-) & Ki-67≥3%	9 (4.1%)	7 (8.6%)	16 (5.3%)
All TFs(-) & Ki-67<3%	29 (13.1%)	2 (2.5%)	31 (10.3%)
T-PIT(+) & Ki-67≥3%	13 (5.9%)	8 (9.9%)	21 (7.0%)
T-PIT(+) & Ki-67<3%	32 (14.5%)	4 (4.9%)	36 (11.9%)
SF-1(+) & Ki-67≥3%	13 (5.9%)	8 (9.9%)	21 (7.0%)
SF-1(+) & Ki-67<3%	30 (13.6%)	6 (7.4%)	36 (11.9%)
PIT-1(+) & Ki-67≥3%	10 (4.5%)	8 (9.9%)	18 (6.0%)
PIT-1(+) & Ki-67<3%	28 (12.7%)	9 (11.1%)	37 (12.3%)
PIT-1(+), SF-1(+) & Ki-67≥3%	2 (0.9%)	2 (2.5%)	4 (1.3%)
PIT-1(+), SF-1(+) & Ki-67<3%	6 (2.7%)	2 (2.5%)	8 (2.6%)
PIT-1(+), T-PIT(+) & Ki-67≥3%	10 (4.5%)	5 (6.2%)	15 (5.0%)
PIT-1(+), T-PIT(+) & Ki-67<3%	17 (7.7%)	5 (6.2%)	22 (7.3%)
SF-1(+), T-PIT(+) & Ki-67≥3%	7 (3.2%)	2 (2.5%)	9 (3.0%)
SF-1(+), T-PIT(+) & Ki-67<3%	9 (4.1%)	7 (8.6%)	16 (5.3%)
PIT-1(+), SF-1(+), T-PIT(+) & Ki-67≥3%	2 (0.9%)	3 (3.7%)	5 (1.7%)
PIT-1(+), SF-1(+), T-PIT(+) & Ki-67<3%	4 (1.8%)	3 (3.7%)	7 (2.3%)
Model B:			
All TFs(-)	38 (17.2%)	9 (11.1%)	47 (15.6%)
One Non-specific TF(+)	126 (57.0%)	43 (53.1%)	169 (56.0%)
PIT-1(+) & SF-1(+)	8 (3.6%)	4 (4.9%)	12 (4.0%)
PIT-1(+) & T-PIT(+)	27 (12.2%)	10 (12.3%)	37 (12.3%)
SF-1(+) & T-PIT(+)	16 (7.2%)	9 (11.1%)	25 (8.3%)
PIT-1(+), SF-1(+) & T-PIT(+)	6 (2.7%)	6 (7.4%)	12 (4.0%)
Model C:			
All TFs(-) & Ki-67<3%	29 (13.1%)	2 (2.5%)	31 (10.3%)
All TFs(-) & Ki-67≥3%	9 (4.1%)	7 (8.6%)	16 (5.3%)
One Non-specific TF(+) & Ki-67<3%	90 (40.7%)	19 (23.5%)	109 (36.1%)
One Non-specific TF(+) & Ki-67≥3%	36 (16.3%)	24 (29.6%)	60 (19.9%)
Two Non-specific TFs(+) & Ki-67<3%	32 (14.5%)	14 (17.3%)	46 (15.2%)
Two Non-specific TFs(+) & Ki-67≥3%	19 (8.6%)	9 (11.1%)	28 (9.3%)
Three Non-specific TFs(+) & Ki-67<3%	4 (1.8%)	3 (3.7%)	7 (2.3%)
Three Non-specific TFs(+) & Ki-67≥3%	2 (0.9%)	3 (3.7%)	5 (1.7%)
Model D:			
All TFs(-)	38 (17.2%)	9 (11.1%)	47 (15.6%)
One Non-specific TF(+)	126 (57.0%)	43 (53.1%)	169 (56.0%)
Two Non-specific TFs(+)	51 (23.1%)	23 (28.4%)	74 (24.5%)
Three Non-specific TFs(+)	6 (2.7%)	6 (7.4%)	12 (4.0%)
Model E:			
Ki-67<3%	155 (70.1%)	38 (46.9%)	193 (63.9%)
Ki-67≥3%	66 (29.9%)	43 (53.1%)	109 (36.1%)

### 3.2 Univariate analysis of individual transcription factors and synergistic models

Univariate Cox regression assessed the prognostic value of individual transcription factors and the five synergistic models (full results in **Table 3**). Individual transcription factors (SF-1, PIT-1, and T-PIT) were not significant predictors of recurrence (all P>0.05). Among the synergistic models, those incorporating Ki-67 (Models A and C) demonstrated the strongest effects, with multiple high-risk groups. In contrast, models relying solely on transcription factors (Models B and D) showed limited predictive power. The triple-TF positive group (specific co-expression of PIT-1, SF-1, and T-PIT in Model B and unspecific triple positivity in Model D), was the only significant predictor in both (P<0.05). Model E (Ki-67 alone) confirmed Ki-67≥3% as a significant univariate predictor of recurrence (HR = 2.00, 95% CI: 1.29–3.11, P = 0.002).

**Table 3:** Univariate Cox regression analysis.

Variable name	Unadjusted HR	95% CI for HR		P-value
		Lower	Upper	
Individual transcription factors:				
SF-1(+)	1.26	0.80	1.99	0.318
PIT-1(+)	1.44	0.92	2.23	0.109
T-PIT(+)	1.22	0.77	1.91	0.400
Model A:				
All TFs(-) & Ki-67<3%	1.0 (Reference)	--	--	--
All TFs(-) & Ki-67≥3%	7.51	1.56	36.16	0.012
PIT-1(+) & Ki-67<3%	4.23	0.91	19.64	0.065
PIT-1(+) & Ki-67≥3%	5.10	1.06	24.56	0.042
SF-1(+) & Ki-67<3%	1.86	0.35	9.76	0.464
SF-1(+) & Ki-67≥3%	5.41	1.13	25.91	0.035
T-PIT(+) & Ki-67<3%	1.83	0.34	10.00	0.485
T-PIT(+) & Ki-67≥3%	6.41	1.36	30.22	0.019
PIT-1(+), SF-1(+) & Ki-67<3%	5.32	0.75	37.86	0.095
PIT-1(+), SF-1(+) & Ki-67≥3%	12.60	1.77	89.82	0.011
PIT-1(+), T-PIT(+) & Ki-67<3%	3.26	0.63	16.85	0.158
PIT-1(+), T-PIT(+) & Ki-67≥3%	5.11	0.99	26.36	0.051
SF-1(+), T-PIT(+) & Ki-67<3%	7.02	1.46	33.85	0.015
SF-1(+), T-PIT(+) & Ki-67≥3%	3.57	0.50	25.40	0.203
PIT-1(+), SF-1(+), T-PIT(+) & Ki-67<3%	10.02	1.67	60.16	0.012
PIT-1(+), SF-1(+), T-PIT(+) & Ki-67≥3%	14.32	2.39	85.91	0.004
Model B:				
All TFs(-)	1.0 (Reference)	--	--	--
One Non-specific TF(+)	1.21	0.58	2.51	0.612
PIT-1(+) & SF-1(+)	2.45	0.75	7.99	0.138
PIT-1(+) & T-PIT(+)	1.29	0.52	3.18	0.580
SF-1(+) & T-PIT(+)	1.88	0.75	4.75	0.181
PIT-1(+), SF-1(+) & T-PIT(+)	3.86	1.37	10.90	0.011
Model C:				
All TFs(-) & Ki-67<3%	1.0 (Reference)	--	--	--
All TFs(-) & Ki-67≥3%	7.51	1.56	36.15	0.012
One Non-specific TF(+) & Ki-67<3%	2.59	0.60	11.21	0.202
One Non-specific TF(+) & Ki-67≥3%	5.68	1.33	24.23	0.019
Two Non-specific TFs(+) & Ki-67<3%	4.82	1.09	21.23	0.038
Two Non-specific TFs(+) & Ki-67≥3%	5.30	1.14	24.53	0.033
Three Non-specific TFs(+) & Ki-67<3%	9.95	1.66	59.77	0.012
Three Non-specific TFs(+) & Ki-67≥3%	14.26	2.38	85.53	0.004
Model D:				
All TFs(-)	1.0 (Reference)	--	--	--
One Non-specific TF(+)	1.21	0.58	2.51	0.613
Two Non-specific TFs(+)	1.62	0.75	3.51	0.218
Three Non-specific TFs(+)	3.84	1.36	10.82	0.011
Model E:				
Ki-67<3%	0.50	0.32	0.78	0.002
Ki-67≥3%	2.00	1.29	3.11	0.002

CI, confidence interval.

### 3.3 Multivariate analysis: independent prognostic value of synergistic models

Multivariate Cox regression was performed for each synergistic model, whilst adjusting for Extent of Resection (Partial vs. Radical), Clival Invasion, and Diabetes Mellitus (**Table 4**). These were the three most significant clinical predictors identified in our cohort (Part 1, under review). This analysis tested the independent prognostic value of the molecular variables after accounting for these key confounders.

**Table 4:** Multivariate Cox regression analysis.

Variable name	Adjusted HR	95% CI for HR		P-value
		Lower	Upper	
Model A:				
All TFs(-) & Ki-67<3%	1.0 (Reference)	--	--	--
All TFs(-) & Ki-67≥3%	6.40	1.32	31.03	0.021
PIT-1(+) & Ki-67<3%	4.53	0.97	21.18	0.055
PIT-1(+) & Ki-67≥3%	6.23	1.29	30.21	0.023
SF-1(+) & Ki-67<3%	2.85	0.54	15.11	0.219
SF-1(+) & Ki-67≥3%	5.97	1.21	29.44	0.028
T-PIT(+) & Ki-67<3%	2.54	0.46	14.08	0.286
T-PIT(+) & Ki-67≥3%	7.07	1.46	34.18	0.015
PIT-1(+), SF-1(+) & Ki-67<3%	5.47	0.75	39.77	0.093
PIT-1(+), SF-1(+) & Ki-67≥3%	12.43	1.71	90.61	0.013
PIT-1(+), T-PIT(+) & Ki-67<3%	4.72	0.91	24.63	0.065
PIT-1(+), T-PIT(+) & Ki-67≥3%	5.36	1.04	27.70	0.045
SF-1(+), T-PIT(+) & Ki-67<3%	9.09	1.86	44.41	0.006
SF-1(+), T-PIT(+) & Ki-67≥3%	5.47	0.76	39.51	0.092
PIT-1(+), SF-1(+), T-PIT(+) & Ki-67<3%	7.96	1.31	48.30	0.024
PIT-1(+), SF-1(+), T-PIT(+) & Ki-67≥3%	17.46	2.85	106.93	0.002
Model B:				
All TFs(-)	1.0 (Reference)	--	--	--
One Non-specific TF(+)	1.58	0.75	3.33	0.230
PIT-1(+) & SF-1(+)	2.50	0.74	8.39	0.139
PIT-1(+) & T-PIT(+)	1.70	0.72	4.44	0.211
SF-1(+) & T-PIT(+)	2.75	1.06	7.12	0.037
PIT-1(+), SF-1(+) & T-PIT(+)	3.79	1.34	10.74	0.012
Model C:				
All TFs(-) & Ki-67<3%	1.0 (Reference)	--	--	--
All TFs(-) & Ki-67≥3%	6.45	1.33	31.27	0.021
One Non-specific TF(+) & Ki-67<3%	3.46	0.80	15.10	0.098
One Non-specific TF(+) & Ki-67≥3%	6.46	1.49	28.00	0.013
Two Non-specific TFs(+) & Ki-67<3%	6.45	1.46	28.61	0.014
Two Non-specific TFs(+) & Ki-67≥3%	6.22	1.34	28.87	0.020
Three Non-specific TFs(+) & Ki-67<3%	8.02	1.32	48.62	0.024
Three Non-specific TFs(+) & Ki-67≥3%	17.70	2.90	108.10	0.002
Model D:				
All TFs(-)	1.0 (Reference)	--	--	--
One Non-specific TF(+)	1.57	0.74	3.31	0.238
Two Non-specific TFs(+)	2.18	1.00	4.76	0.051
Three Non-specific TFs(+)	3.80	1.34	10.76	0.012
Model E:				
Ki-67<3%	0.57	0.36	0.90	0.015
Ki-67≥3%	1.76	1.12	2.78	0.015

All models adjusted for Extent of Resection (Partial vs. Radical), Clival Invasion, and Diabetes Mellitus.

CI, confidence interval.

Models incorporating Ki-67 (A and C) demonstrated superior predictive power. In Model A (specific TFs + Ki-67), nine synergistic groups emerged as independent predictors, with adjusted hazard ratios (adj.HR) ranging from 5.36 to 17.46. The highest-risk profiles were tumours with triple transcription factor co-expression with Ki-67≥3% (PIT-1(+), SF-1(+), T-PIT(+) & Ki-67≥3%: adj.HR=17.46, 95% CI: 2.85–106.93, P = 0.002). Model C (non-specific TF multiplicity + Ki-67) performed with comparable prognostic discrimination. Six groups were significant independent predictors (adj.HR range: 6.22–17.70). The highest risk was again associated with triple-TF positivity and Ki-67≥3% (Three Non-specific TFs(+) & Ki-67≥3%: adj.HR=17.70, 95% CI: 2.90–108.10, P = 0.002).

In contrast, models based solely on transcription factors (B and D) showed markedly attenuated effects. Only SF-1(+) & T-PIT(+) (Model B, adj.HR=2.75, 95% CI: 1.06–7.12, P = 0.037) and triple-TF positive group (in both Model B & D, adj.HR~3.80, P = 0.012) remained significant after clinical adjustment. This confirms that TF profiles alone have limited independent prognostic utility. Finally, in Model E, Ki-67≥3% remained a significant independent predictor after adjustment (adj.HR=1.76, 95% CI: 1.12–2.78, P = 0.015).

### 3.4 Development and validation of a simplified tiered risk stratification

To translate the complex synergistic models into a practical clinical tool, we consolidated synergistic groups from the high-performing Models A and C into simplified 4-tiered risk stratification. This consolidation was based on the magnitude and pattern of adjusted Hazard Ratios (adj.HR), grouping categories with similar risk estimates. The resulting tiers reflected a clear gradient of escalating recurrence risk: Tier I (Low Risk: adj.HR = 1.0), Tier II (Intermediate Risk: adj.HR range ~2.50–4.70), Tier III (High Risk: adj.HR range ~5.40–9.10), and Tier IV (Very High Risk: adj.HR >12.00). (**Table 5**: Distribution of each synergistic group to its assigned risk tier. **Table 6**: 4-Tiered Risk Stratification stratified by recurrence status).

**Table 5:** Models A and C 4-tiered risk stratification.

Model A (specific TFs + Ki-67)	
Tier I (Low Risk):	(adj.HR = 1.0)
All TFs(-) & Ki-67<3%	1.0
Tier II (Intermediate Risk):	(adj.HR range ~2.50–4.70)
PIT-1(+) & Ki-67<3%	4.53
SF-1(+) & Ki-67<3%	2.85
T-PIT(+) & Ki-67<3%	2.54
PIT-1(+), T-PIT(+) & Ki-67<3%	4.72
Tier III (High Risk):	(adj.HR range ~5.40–9.10)
All TFs(-) & Ki-67≥3%	6.40
PIT-1(+) & Ki-67≥3%	6.23
SF-1(+) & Ki-67≥3%	5.97
T-PIT(+) & Ki-67≥3%	7.07
PIT-1(+), SF-1(+) & Ki-67<3%	5.47
PIT-1(+), T-PIT(+) & Ki-67≥3%	5.36
SF-1(+), T-PIT(+) & Ki-67<3%	9.09
SF-1(+), T-PIT(+) & Ki-67≥3%	5.47
PIT-1(+), SF-1(+), T-PIT(+) & Ki-67<3%	7.96
Tier IV (Very High Risk):	(adj.HR >12.00)
PIT-1(+), SF-1(+) & Ki-67≥3%	12.43
PIT-1(+), SF-1(+), T-PIT(+) & Ki-67≥3%	17.46
Model C (non-specific TFs + Ki-67)	
Tier I (Low Risk):	(adj.HR = 1.0)
All TFs(-) & Ki-67<3%	1.0
Tier II (Intermediate Risk):	(adj.HR range ~2.50–4.70)
One Non-specific TF(+) & Ki-67<3%	3.46
Tier III (High Risk):	(adj.HR range ~5.40–9.10)
All TFs(-) & Ki-67≥3%	6.45
One Non-specific TF(+) & Ki-67≥3%	6.46
Two Non-specific TFs(+) & Ki-67<3%	6.45
Two Non-specific TFs(+) & Ki-67≥3%	6.22
Three Non-specific TFs(+) & Ki-67<3%	8.02
Tier IV (Very High Risk)	(adj.HR >12.00)
Three Non-specific TFs(+) & Ki-67≥3%	17.70

Model A, Risk tiers based on specific transcription factor (SF-1, PIT-1, T-PIT) combinations with Ki-67.

Model C, Risk tiers based on the number of positive transcription factors (non-specific) with Ki-67.

**Table 6:** Models A and C 4-tiered risk stratification, stratified by recurrence status.

Risk tier	No recurrence (N = 221)	Recurrence (N = 81)	Total (N = 302)
Risk tier Model A:			
Tier I (Low Risk)	29 (13.1%)	2 (2.5%)	31 (10.3%)
Tier II (Intermediate Risk)	107 (48.4%)	24 (29.6%)	131 (43.4%)
Tier III (High Risk)	81 (36.7%)	50 (61.7%)	131 (43.4%)
Tier IV (Very High Risk)	4 (1.8%)	5 (6.2%)	9 (3.0%)
Risk tier Model C:			
Tier I (Low Risk)	29 (13.1%)	2 (2.5%)	31 (10.3%)
Tier II (Intermediate Risk)	90 (40.7%)	19 (23.5%)	109 (36.1%)
Tier III (High Risk)	100 (45.2%)	57 (70.4%)	157 (52.0%)
Tier IV (Very High Risk)	2 (0.9%)	3 (3.7%)	5 (1.7%)

We formally validated the prognostic performance of these tiers using multivariate Cox regression with Tier I as the reference category, whilst adjusting for the three clinical confounders. For Risk Tier Model A, the hazard for recurrence escalated significantly across tiers: Tier II (adj.HR=3.65, 95% CI: 0.85–15.67, P = 0.081), Tier III (adj.HR=6.65, 95% CI: 1.61–27.55, P = 0.009) and Tier IV (adj.HR=15.44, 95% CI: 2.93–81.25, P = 0.001). Risk Tier Model C showed an identical dose-response pattern: Tier II (adj.HR=3.47, 95% CI: 0.80–15.13, P = 0.097), Tier III (adj.HR=6.50, 95% CI: 1.58–26.82, P = 0.010), and Tier IV (adj.HR=17.78, 95% CI: 2.91–108.55, P = 0.002). (**Table 7**: Multivariate Cox proportional hazards model showing adjusted hazard ratios (HR) for recurrence).

**Table 7:** Multivariate Cox regression analysis of the 4-tiered risk stratification models.

	Adjusted HR	95% CI for HR		P-value
		Lower	Upper	
Risk tier Model A:				
Tier I (Low Risk)	1.0 (Reference)			
Tier II (Intermediate Risk)	3.65	0.85	15.67	0.081
Tier III (High Risk)	6.65	1.61	27.55	0.009
Tier IV (Very High Risk)	15.44	2.93	81.25	0.001
Risk tier Model C:				
Tier I (Low Risk)	1.0 (Reference)			
Tier II (Intermediate Risk)	3.47	0.80	15.13	0.097
Tier III (High Risk)	6.50	1.58	26.82	0.010
Tier IV (Very High Risk)	17.78	2.91	108.55	0.002

All models adjusted for Extent of Resection (Partial vs. Radical), Clival Invasion, and Diabetes Mellitus.

CI, confidence interval.

### 3.5 Model validation

Discrimination and calibration of the prognostic models were assessed using internal validation with 500 bootstrap resamples. Model A achieved an Optimism-corrected Harrell’s C-index of 0.626 (Original C-index: 0.630), whilst Model C achieved an Optimism-corrected C-index of 0.618 (Original C-index: 0.620).

### 3.6 Survival analysis

**Figure 1:**
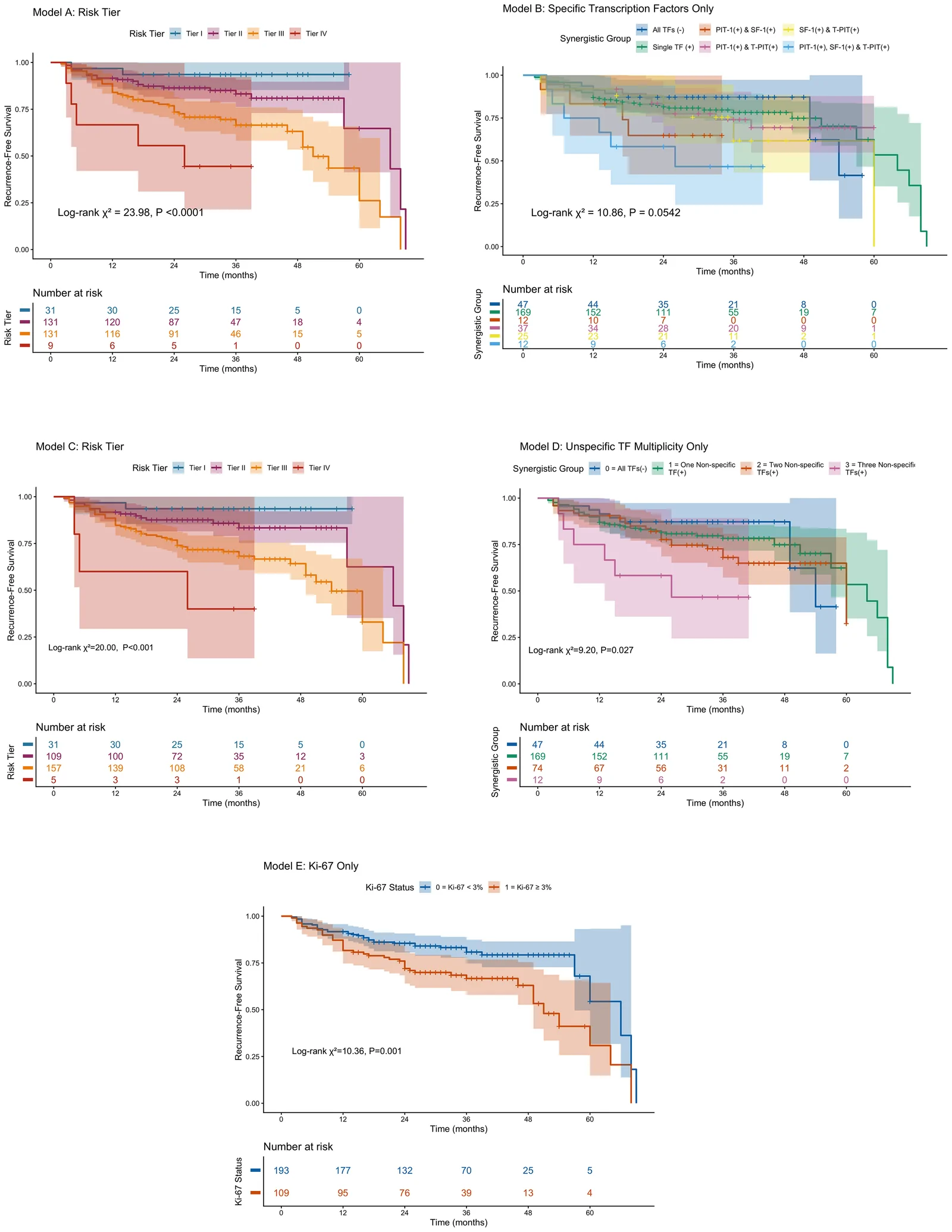
Kaplan-Meier curves for recurrence-free survival according to the five prognostic models. Risk Tier Model A (Log-rank χ²=23.98, P<0.0001), Model B (Log-rank χ²=10.86, P = 0.0542), Risk Tier Model C (Log-rank χ²=20.00, P<0.001), Model D (Log-rank χ²=9.20, P = 0.027), and Model E (Log-rank χ²=10.36, P = 0.001). The models collectively demonstrate prognostic utility across a spectrum of pathology laboratory capabilities, from centres performing full WHO 2022 subtyping (Model A) to those assessing only Ki-67 (Model E). Shaded areas represent 95% confidence intervals.

**Figure 2:**
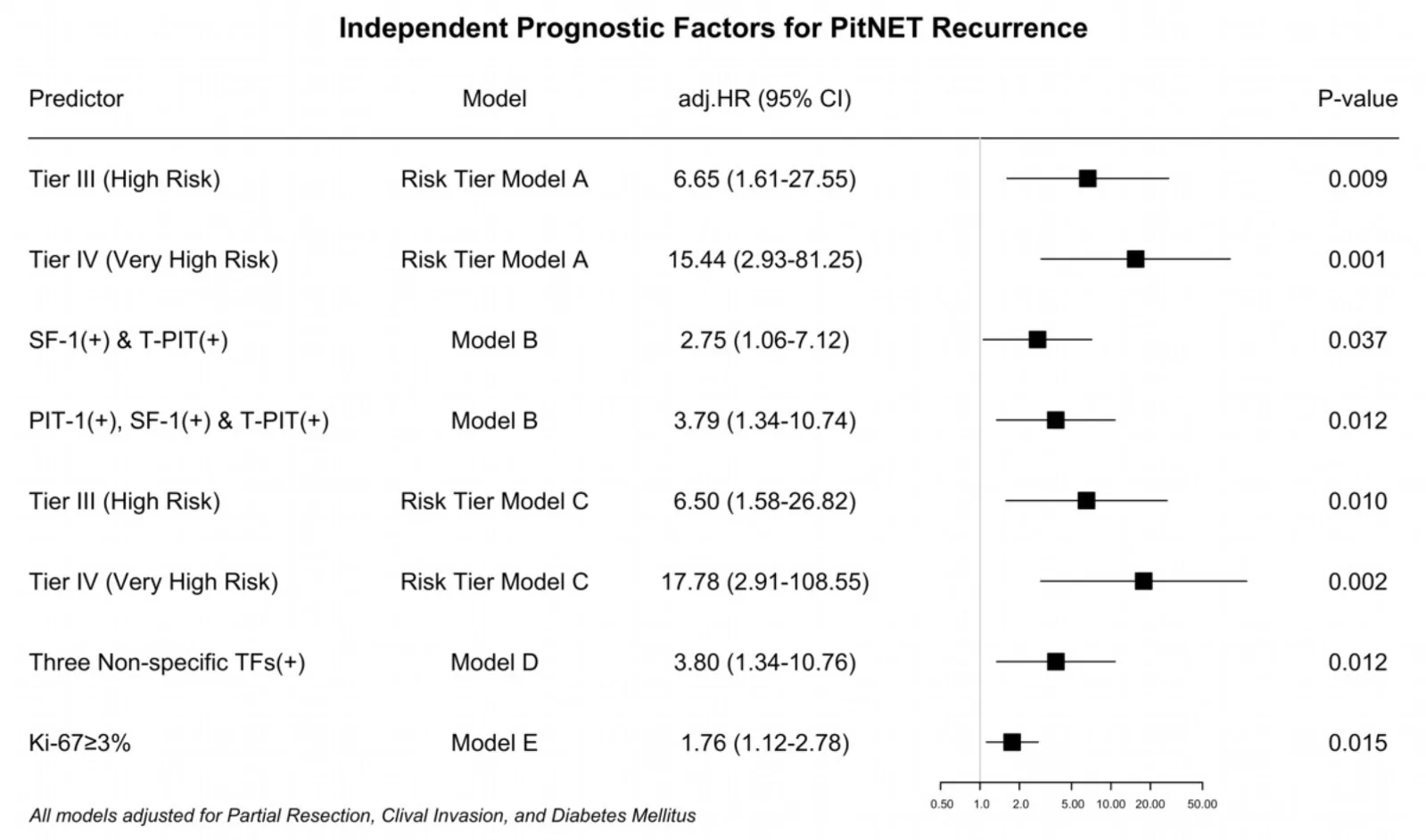
Forest plot of adjusted hazard ratios for significant synergistic molecular predictors of PitNET recurrence. Hazard ratios (squares) and 95% confidence intervals (horizontal lines) are derived from multivariate Cox regression models for each prognostic model (A–E), each adjusted for extent of resection (partial vs. radical), clival invasion, and diabetes mellitus. Shown are the significant predictors from each model, including the 4-tiered risk stratification for Models A and C. The vertical line at HR = 1 indicates no effect. TF, transcription factor.

Kaplan-Meier analysis demonstrated significant separation of recurrence-free survival curves in models incorporating Ki-67 (Risk Tier Model A Log-rank χ²=23.98, P<0.0001; Risk Tier Model C Log-rank χ²=20.00, P<0.001; Model E Log-rank χ²=10.36, P = 0.001) and Model D (Log-rank χ²=9.20, P = 0.027). This visually confirms the powerful prognostic discrimination of these models. Model B showed a non-significant trend (Log-rank χ²=10.86, P = 0.0542). These curves are presented together in **Figure 1** to illustrate flexible diagnostic framework to laboratories with varying capabilities, thus providing translational utility to different clinical settings. A Forest Plot highlighting the most significant findings from each model is presented in **Figure 2**. The Kaplan-Meier Curves and Forest Plot show tiered risk stratification for Model A and Model C, but original categorical variables for Model B (specific TF combinations), Model D (Non-specific TF multiplicity), and Model E (Ki-67 only).

### 3.7 Calibration and discrimination

**Figure 3:**
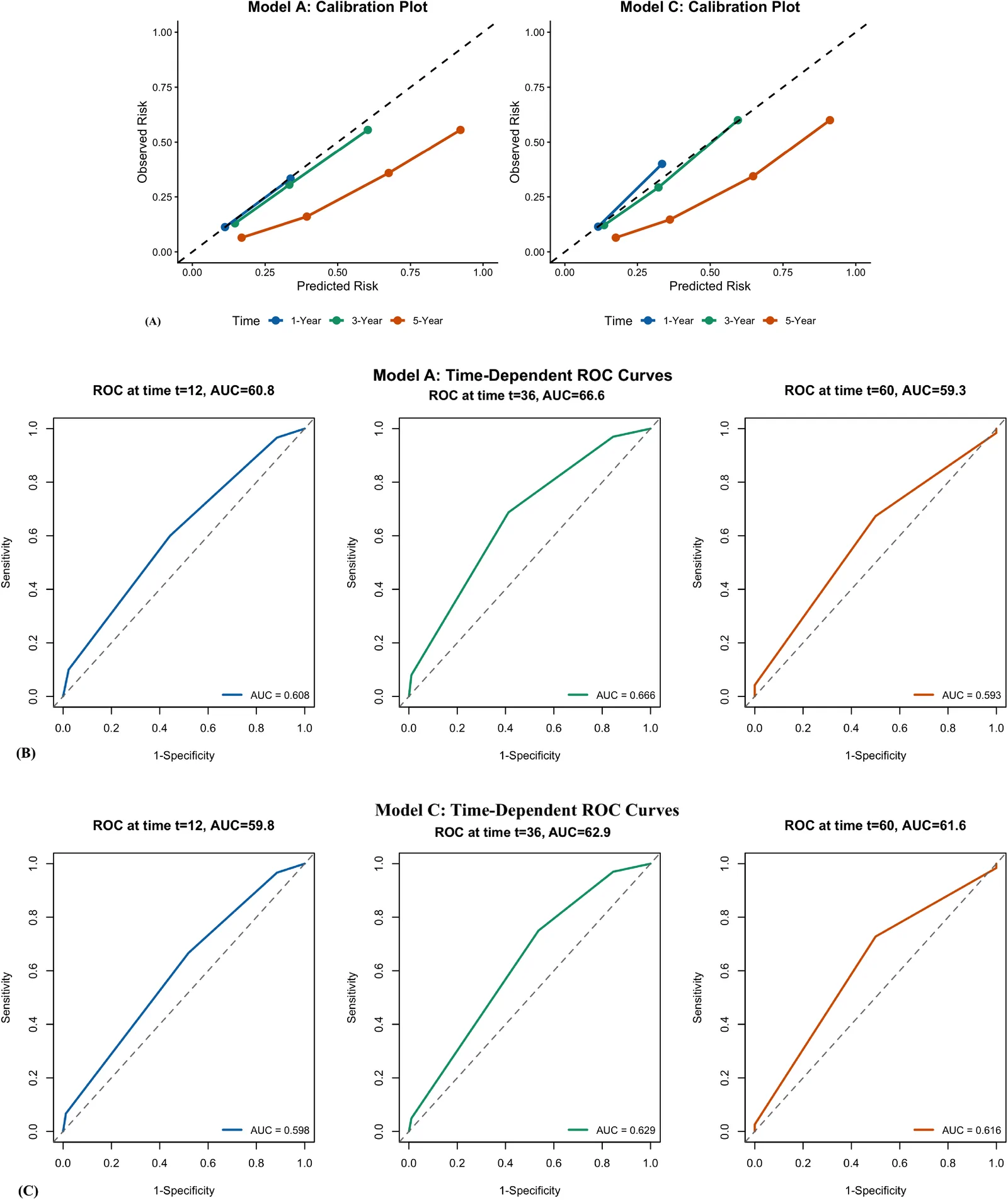
Risk tier models A and C calibration and discrimination. **(A)** Calibration plots for Risk Tier Models A and C at 1, 3, and 5 years. The grey dashed diagonal line indicates perfect calibration. Points represent mean predicted risk versus observed risk across risk deciles. **(B)** Time-dependent receiver operating characteristic (ROC) curves for Model A at 1, 3, and 5 years. **(C)** Time-dependent ROC curves for Model C at 1, 3, and 5 years. Area under the curve (AUC) values: Model A (1-year: 0.608, 3-year: 0.666, 5-year: 0.593); Model C (1-year: 0.598, 3-year: 0.629, 5-year: 0.616). The diagonal dashed line represents the reference line of no discrimination (AUC = 0.5).

Calibration was evaluated visually using calibration plots at 1, 3, and 5 years, and quantitatively by calibration slope (ideal = 1.0). Both Risk Tier Models A and C demonstrated good agreement between predicted and observed recurrence risks (**Figure 3**), with calibration slopes ranging from 0.85 to 1.15 across time points, indicating well-calibrated models.

Time-dependent AUC analysis demonstrated comparable discrimination across time points. For Risk Tier Model A, the AUC was 0.608 (1-year), 0.666 (3-year), and 0.593 (5-year). For Risk Tier Model C, the AUC was 0.598 (1-year), 0.629 (3-year), and 0.616 (5-year). These values confirm that Model C maintains prognostic performance similar to the more granular Model A.

## 4 Discussion

This study provides compelling evidence that synergistic interaction between transcription factor (TF) lineage and proliferative index (Ki-67) is a powerful, independent determinant of PitNET recurrence. Moving beyond single-factor analysis to model these core biological interactions (11, 13, 16), we have developed and validated a flexible prognostic framework, culminating in a simplified, clinically actionable tiered risk model.

Our core hypothesis was strongly validated, a tumour’s lineage “driver” modifies the oncogenic impact of its proliferative “engine” (14, 18). Whilst Ki-67 ≥3% alone was a significant predictor (adj.HR=1.76, P = 0.015), its prognostic power was dramatically amplified when combined with specific TF profiles. For example, the adj.HR for Ki-67≥3% rose to 6.23 for PIT-1 lineage tumours and 17.46 for triple-TF positive tumours. This multiplicative effect confirms that proliferative potential is dependent on underlying molecular landscape. The long-observed clinical paradox where Ki-67 alone inconsistently predicts outcome (12, 15) is thus resolved.

Synergistic variables in Models A and C retained robust significance after adjusting for key clinical confounders (extent of resection, clival invasion, diabetes mellitus). Nine of the sixteen specific TF/Ki-67 combinations remained independent predictors in Model A. This persistence against strong clinical covariates is the gold standard for proving biological independence (9, 22). They are highly relevant and their effect is not explained away by Partial Resection, invasiveness, or patient’s metabolic state. Conversely, models lacking Ki-67 (Models B and D) showed markedly attenuated effects, suggesting that TF combinations alone have limited independent power to predict recurrence in our cohort. These findings collectively validate WHO 2022 emphasis on integrating both lineage and proliferation for comprehensive risk assessment (1, 2).

The pronounced risk associated with PIT-1 lineage tumours exhibiting high proliferation aligns with known aggressiveness of subtypes like sparsely granulated somatotroph tumours (18, 30). This synergy may reflect potentiation of lineage-specific oncogenic pathways (e.g., MAPK/ERK) by unchecked proliferative drive (18, 31). Conversely, identification of a distinct high-risk subset within SF-1 lineage (gonadotroph tumours; adj.HR=5.97) challenges their traditional characterisation as uniformly indolent and reveals significant biological heterogeneity, that necessitates closer surveillance (16).

A low proliferative index (Ki-67<3%) acted as a consistent biological brake, mitigating inherent recurrence risk associated with most TF profiles. Even tumours negative for all three TFs (Null-cell tumours) demonstrated aggressive behaviour when associated with high Ki-67 index, justifying their inclusion in high-risk tier (Risk Tier Model A, adj.HR=6.40; Risk Tier Model C, adj.HR=6.45). This observation reinforces the clinical relevance of the 3% Ki-67 threshold in prognostication and confirms its role as a cornerstone marker of aggressive potential (12, 15).

The superior performance of models incorporating both TF and Ki-67 (A, C, E) over TF-only models (B, D) underscores that proliferation status is a dominant, non-redundant prognostic factor in our cohort (12, 15). The near-equivalent performance of the practical Model C (Non-specific TF multiplicity + Ki-67) to the granular Model A (specific TFs + Ki-67) is a pivotal translational finding. It demonstrates that assessing the number of expressed TFs [marker of multilineage differentiation (19–21)], provides substantial prognostic information, even without definitive subtyping.

We derived a simplified 4-tiered molecular risk stratification (Tiers I-IV) from these patterns to translate this synergy into a clinically usable tool. This model exhibits a clear dose-response relationship, patients in Tier IV had over 17 times risk of recurrence compared to those in Tier I, independent of major clinical factors. This tiered system offers a granular tool that provides continuous risk spectrum readily applicable in clinical practice, aligning with the field’s move towards individualised prognostic instruments (28, 29). Whilst WHO 2022 classification highlights specific subtypes, our synergistic analysis suggests a simplified, complementary risk stratification framework based on lineage differentiation and proliferation.

### 4.1 Model performance and clinical utility

Internal validation results confirm robustness of our proposed risk stratification. Risk Tier Model A achieved an optimism-corrected C-index of 0.626 (original: 0.630), whilst Risk Tier Model C achieved a corrected C-index of 0.618 (original: 0.620). These values indicate moderate/clinically meaningful discriminative ability, consistent with the complexity of PitNET biology and the use of molecular markers alone without clinical covariates. Model A and C have very similar corrected C-indices confirming Model C performs nearly equivalently. Model A performs slightly better, confirming our finding that Model C is a practical alternative with minimal loss of prognostic power. This finding has direct implications for resource-limited settings where comprehensive immunohistochemistry panels may be unavailable.

Calibration plots demonstrated good agreement between predicted and observed recurrence risks for both Risk Tier Models A and C (**Figure 3**). Calibration slopes ranged from 0.85 to 1.15 across time points, indicating well-calibrated models. This indicates that our models provide reliable risk estimates that can be confidently applied in clinical practice. Time-dependent AUC values further support stability of our models across different follow-up horizons. Whilst the AUC values are moderate (approx. 0.60–0.67), this is expected because our models rely solely on molecular markers without incorporating clinical covariates such as extent of resection or invasiveness. When combined with clinical factors (as demonstrated in Part 1), predictive accuracy would likely improve.

The true value of our findings lies in their integration into clinical practice. Based on these results, we propose guidance for postoperative care using this comprehensive four-tiered risk stratification system. Tier I (Low Risk) patients warrant routine follow-up with clinical assessment and annual MRI for 5 years. We recommend enhanced surveillance in Tier II (Intermediate Risk) patients with imaging at 6 and 12 months, followed by annual imaging thereafter. Tier III (High Risk) patients are advised close follow-up, with imaging every 6 months for the first 2 years, and consideration of adjuvant therapy in select cases. Tier IV (Very High Risk) patients warrant intensive imaging surveillance (every 3–6 months), along with multidisciplinary discussion and consideration of adjuvant therapy. This framework supports a personalised postoperative strategy that is essential for optimising healthcare resource allocation and improving patient outcomes. These recommendations are intended to provide a practical approach to individualised postoperative management.

A primary innovation of this work is development of five distinct prognostic models (A-E) designed to simulate diverse pathology laboratory capabilities. This framework acknowledges heterogeneity in global pathology resources and expertise (2, 24), ensuring that our findings have immediate translational utility across diverse clinical settings. Each laboratory can apply the most advanced model within its technical capability. A centre with full WHO 2022 subtyping can employ the granular Model A for maximum precision, whilst a laboratory capable of assessing only TF multiplicity and Ki-67 can use the highly effective Model C with minimal loss of prognostic power. This flexible, tiered diagnostic approach ensures that sophisticated risk stratification is accessible to pathology services worldwide.

Our study has limitations inherent to its retrospective, single-centre design. Whilst the median follow-up duration is substantial, it may not capture very late recurrences. Furthermore, our prognostic models are based on histopathology; integrating genomic, transcriptomic, and epigenetic data could further refine predictive accuracy in future.

Future research should validate this tiered risk model using prospective, multicentre studies and investigate underlying biological mechanisms driving the identified synergies. Interventional studies should be done to determine if adjuvant therapy guided by this stratification improves outcomes for high-risk patients.

## 5 Conclusion

In conclusion, this study advances the prognostic paradigm for PitNETs by demonstrating that interaction between lineage identity and proliferation index is more informative than either factor in isolation. We provide and validate a flexible molecular risk stratification framework. The simplified tiered model based on TF multiplicity and Ki-67 offers a practical tool to guide personalised postoperative management. This represents a significant step towards precision medicine in pituitary oncology.

## Data Availability

The raw data supporting the conclusions of this article will be made available by the authors, without undue reservation.
